# B cell depletion therapy upregulates Dkk-1 skin expression in patients with systemic sclerosis: association with enhanced resolution of skin fibrosis

**DOI:** 10.1186/s13075-016-1017-y

**Published:** 2016-05-21

**Authors:** Dimitrios Daoussis, Athanassios Tsamandas, Ioannis Antonopoulos, Alexandra Filippopoulou, Dionysios J. Papachristou, Nicholaos I. Papachristou, Andrew P. Andonopoulos, Stamatis-Nick Liossis

**Affiliations:** Division of Rheumatology, Department of Internal Medicine, Patras University Hospital, University of Patras Medical School, Rion, Patras, 26504 Greece; Department of Patholology, Patras University Hospital, University of Patras Medical School, Patras, Greece; Department of Anatomy-Histology-Embryology, Laboratory of Bone and Soft Tissue Studies, University of Patras Medical School, Patras, Greece

**Keywords:** Systemic sclerosis, Scleroderma, Rituximab, B cell depletion, Dickkopf-1, Dkk-1, TGFβ, Fibrosis, Skin

## Abstract

**Background:**

Rituximab (RTX) may favorably affect skin and lung fibrosis in patients with systemic sclerosis (SSc); however, the underlying molecular mechanisms remain unknown. We aimed to explore the hypothesis that RTX may mediate its antifibrotic effects by regulating the expression of Dickkopf-1 (Dkk-1), an inhibitor of the Wnt pathway.

**Methods:**

Fourteen patients with SSc and five healthy subjects were recruited. Dkk-1 expression was immunohistochemically assessed in skin biopsies obtained from 11 patients with SSc (8 treated with RTX and 3 with standard treatment), whereas DKK1 gene expression was assessed in 3 patients prior to and following RTX administration.

**Results:**

In baseline biopsies obtained from all patients with SSc but not in healthy subjects, Dkk-1 was undetectable in skin fibroblasts. Following RTX treatment, four out of eight patients had obvious upregulation of Dkk-1 skin expression. Similarly, RTX treatment correlated with a significant 4.8-fold upregulation of DKK1 gene expression (*p* = 0.030). In contrast, TGFβ expression in the upper dermis was significantly attenuated following treatment. Moreover, this decreased expression of TGFβ in the skin was significantly more pronounced in the subgroup of patients with Dkk-1 upregulation. In this subgroup TGFβ was downregulated by 50.88 % in contrast to only 15.98 % in patients who did not have Dkk-1 upregulation (*p* = 0.022).

**Conclusions:**

This is the first study demonstrating a link between B cell depletion and skin Dkk-1 upregulation in patients with SSc. RTX-mediated B cell depletion may mechanistically function via the recently established TGFβ-Dkk-1 axis in improving skin fibrosis.

**Electronic supplementary material:**

The online version of this article (doi:10.1186/s13075-016-1017-y) contains supplementary material, which is available to authorized users.

## Background

Systemic sclerosis (SSc) is a complex systemic rheumatic disease characterized by autoimmunity, vasculopathy, and aberrant fibroblast activation, eventually leading to fibrosis, tissue damage, and organ failure [1]. The therapeutic armamentarium for SSc is very restricted; numerous therapies have been tested and either failed or had a modest effect. However, an increasing amount of clinical evidence accumulated over the last few years suggests that B cell depletion therapy may favorably affect skin and lung fibrosis in patients with SSc [2]. We have previously reported that RTX treatment improves skin thickening and lung function in patients with SSc [3]; this effect seems to be further enhanced following long-term treatment [4, 5]. Similar data have been also reported by other research groups [6–8]. Most importantly, recent large-scale multicenter studies, including one from the European League against Rheumatism Scleroderma Trial and Research group, reported encouraging results, thus, supporting the concept of B cell depletion therapy in SSc [9, 10]. It is currently not known how RTX may mediate its potential beneficial effects in SSc. However, experimental evidence indicates that B cells are critically involved in the fibrotic process [11–14].

SSc is a disease characterized by enhanced fibroblast activation leading to increased collagen production and eventually tissue fibrosis. There have been many theories related to the drivers of aberrant fibroblast activation in SSc; recent studies have provided strong experimental data pointing to the direction of the canonical Wnt pathway as a central mediator of the fibrotic process in SSc [15–18]. The Wnt pathway is a developmental pathway with β-catenin serving as the main signaling molecule. The activation of the Wnt pathway is strictly controlled by several soluble inhibitors such as Dickkopf-1 (Dkk-1) [19]. Experimental data indicate that the Wnt pathway is highly activated in the skin in scleroderma. Dkk-1 is virtually undetectable in the skin of patients with SSc in sharp contrast to healthy skin where it is clearly expressed. Moreover, it was found that transforming growth factor β (TGFβ), the most potent profibrotic molecule that is crucially involved in the pathophysiology of SSc, downregulates Dkk-1 expression [20]. These data have unraveled a previously unknown link between TGFβ and the Wnt pathway (TGFβ → downregulation of Dkk-1 → upregulation of the Wnt pathway → fibrosis) and have highlighted the role of Dkk-1 in the fibrotic process.

In our study we aimed to explore the hypothesis that B cell depletion therapy may mediate its antifibrotic effects via the Wnt pathway. More specifically, we aimed to assess (1) expression of Dkk-1 and TGFβ in the skin and circulating levels in patients with SSc prior to and following B cell depletion therapy and (2) DKK1 gene expression in cultured fibroblasts obtained from patients with SSc prior to and following B cell depletion therapy.

We report herein that Dkk-1 is upregulated following B cell depletion therapy in a subset of patients with SSc; these patients exhibit the most profound reduction in skin fibrosis following treatment.

## Methods

### Patients

Fourteen patients with SSc, fulfilling the preliminary American College of Rheumatology criteria for the classification of the disease [21], were recruited. Eleven patients were originally enrolled in a proof of concept study performed in our institution, assessing the clinical efficacy of RTX in SSc [3]. In this study, eight patients were randomized to the treatment arm and received two courses of RTX (at baseline and at 6 months), and six patients were randomized to the control arm and received standard therapy. Skin biopsies from clinically involved skin were obtained from all patients in the treatment group (*n* = 8) and in three patients in the control group (at baseline and at 6 months). Skin biopsies from these 11 patients were immunohistochemically assessed. Three additional patients with SSc treated with RTX, were similarly subjected to skin biopsies at baseline and at 6 months; these biopsies were used for fibroblast extraction and gene expression analysis.

Demographic and clinical characteristics of the study subjects and clinical outcomes following treatment have already been reported in detail elsewhere [3]. Briefly, all patients had diffuse disease, were anti-Scl70 positive, and had no change in medication and/or dosage of treatment administered during the last 12 months before enrollment. Patients in the RTX group had a significant improvement in pulmonary function tests (PFTs) and skin thickening at 1 year (following two courses of RTX treatment) compared to baseline. There was a significant increase in forced vital capacity (FVC) (mean ± SD 68.13 ± 19.69 vs 75.63 ± 19.73, at baseline vs 1 year, respectively, *p =* 0.0018) and diffusing lung capacity for carbon monoxide (DLco) (mean ± SD 52.25 ± 20.71 vs. 62 ± 23.21, at baseline vs 1 year, respectively, *p* = 0.017) in the RTX group. The modified Rodnan skin score (MRSS) also improved significantly in the RTX group compared to the baseline score (mean ± SD, 13.5 ± 6.84 vs 8.37 ± 6.45, at baseline vs 1 year, respectively, *p* < 0.001).

As a disease control group for the current study we used three patients from the control arm of the original study, for whom skin biopsies were available. Two of these patients were receiving cyclophosphamide, whereas the third patient received no treatment. Skin biopsies from five age-matched and sex-matched healthy subjects were evaluated as healthy controls.

The study has been approved by a local Ethics Committee (Patras University Hospital, Patras, Greece) and written informed consent was obtained from all participating individuals.

### Assessment of circulating levels of Dkk-1, TGFβ and IL-6

Circulating Dkk-1, TGFβ and IL-6 levels were measured using a solid phase immunoassay, according to the manufacturer’s instructions (R&D Systems, MN, USA). All measurements were performed in triplicates for each sample and the mean value was calculated.

### Skin histology and immunohistochemistry

Skin biopsies of 5 mm were taken from skin affected by lesions on the forearm. All biopsies were fixed in 10 % neutral buffered formalin and embedded in paraffin. Dkk-1 and TGFβ expression were immunohistochemically assessed using mouse anti-human monoclonal antibodies (R&D Systems, MN, USA); analysis was performed separately for the upper and lower dermis. Masson’s trichrome was used for skin collagen visualization and fibrosis evaluation. Computerized image analysis using the Image J software was used to quantify the results as previously described [3, 22].

### Extraction and culture of skin fibroblasts

Biopsies were mechanically minced using a scalpel and then fibroblasts were extracted using a standard protocol. Fibroblasts were cultured in EMEM supplemented with 10 % FBS and antibiotics (penicillin (100 U/ml), streptomycin (100 mg/ml)); fibroblasts from the third to the fifth passages were used for experiments. In some experiments fibroblasts (obtained from healthy subjects and patients with SSc) were treated with serum obtained from patients with SSc (*n* = 3) prior to and 6 months after treatment with RTX or serum obtained from healthy controls. These experiments were performed to explore whether soluble factors affect DKK1 expression in skin fibroblasts. In these experiments fibroblasts were subjected to 24 hours starvation prior to treatment with 10 % human serum for another 24 hours.

### Fibroblast lysis, RNA extraction and DKK1 gene expression

Cells were detached using trypsin, lysed, and subjected to mRNA extraction according to a standard protocol (RNeasy Mini Kit, Qiagen) following manufacturer’s instructions. RNA purity was confirmed using a NanoDrop Spectrophotometer (Thermo Scientific). cDNA synthesis was carried out using the iScript cDNA synthesis kit (Bio-Rad Laboratories, Hercules, CA, USA) from 1 μg of total RNA. DKK1, Thrombospondin 1 (THBS1) and glyceraldehyde 3-phosphate dehydrogenase (GAPDH) mRNA relative expression levels were assessed, using the iTaq Universal SYBR Green supermix (Bio-Rad Laboratories) with CFX96 Touch Real-time System (Bio-Rad Laboratories). For human DKK1, THBS1 and GAPDH gene-specific KiCqStart™ primers were purchased from Sigma-Aldrich Co. The relative expression level of the gene of interest was calculated with the comparative 2^*ΔΔCΤ*^ method and all samples were normalized to GAPDH. All experiments were independently performed in duplicate three times, each time using 1 μg of template RNA.

### Statistical analysis

Statistical analysis was performed using the GraphPad Prism software version 5. Data are presented as mean ± SEM, median (lower and upper quartile values), or percentages, as appropriate. Student’s *t* test was used for comparisons between groups. Significance was defined as *p* < 0.05 (two-tailed).

## Results

### Dkk-1 deficiency in the skin of patients with scleroderma is restored following B cell depletion therapy

**Fig. 1:**
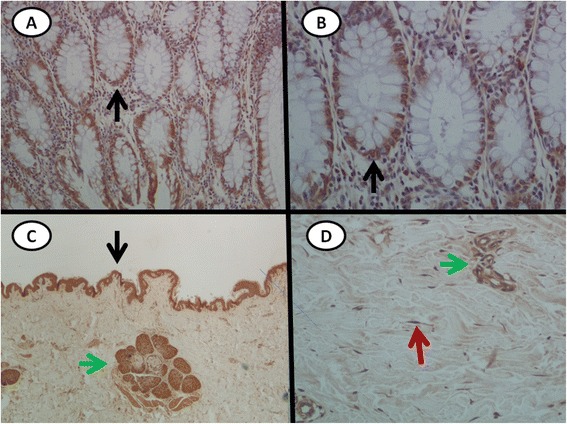
Dickkopf-1 (Dkk-1) is expressed in the normal colon and skin. Dkk-1 expression in a section from a positive control (normal colon) (**a**, **b**). Note the cytoplasmic expression in the basis of crypt epithelium (*black arrows*). Streptavidin biotin peroxidase: magnification × 20 (**a**), × 400 (**b**). Dkk1-expression in a section from normal skin (**c**, **d**). Dkk-1 is clearly expressed at the epidermis (*black arrows*), appendices (*green arrows*) and spindle-like cells (*red arrow*). Streptavidin biotin peroxidase: magnification × 20 (**c**), × 400 (**d**)

Dkk-1 was expressed in the epidermis, appendices, and fibroblasts in the dermis in all the biopsies obtained from healthy subjects. In sharp contrast, in all baseline biopsies obtained from patients with SSc (*n* = 11), Dkk-1 was virtually undetectable in skin fibroblasts. In fact, weak Dkk-1 staining was detected at the epidermis in only two patients (one from the RTX and one from the disease control group). Representative histological analysis of normal skin and the human colon (used as a positive control for Dkk-1 expression) is shown in Fig. 1. These data confirm previously reported results [20].

**Fig. 2:**
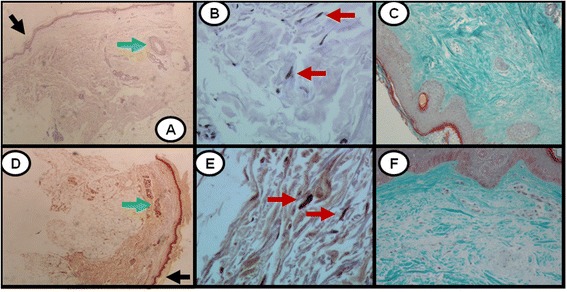
Dickkopf-1 (Dkk-1) is upregulated responders to rituximab (RTX) therapy. In the baseline biopsy (**a**, **b**) Dkk-1 is not expressed in the epidermis (*black arrow*), appendices (*green arrow*), and spindle-like cells (*red arrows*). Following RTX treatment (**d**, **e**) there is clear expression of Dkk-1 in the epidermis (*black arrow*), appendices (*green arrow*) and spindle-like cells (*red arrows*). Streptavidin biotin peroxidase: magnification × 20 (**a**, **d**), × 400 (**b**, **e**). Upregulation of Dkk-1 expression is associated with a decrease in collagen accumulation (prior to (**c**) and after (**f**) RTX treatment). Masson’s trichrome × 100

Following B cell depletion therapy, four out of eight patients had obvious expression of Dkk-1 in skin fibroblasts, in sharp contrast to the disease control group in whom there was no Dkk-1 expression seen in fibroblasts in the follow-up biopsies. Representative histological analysis from a patient showing Dkk-1 upregulation after RTX treatment is shown in Fig. 2. Histological analysis of biopsies from all remaining patients is shown in the Additional files (see Additional file 1: Figures S1-S3 for depiction of patients with Dkk-1 upregulation after RTX treatment, Additional file 2: Figures S4-S6 and Additional file 3: Figure S7 for depiction of patients with no Dkk-1 upregulation after RTX treatment, and Additional file 4: Figures S8-S10 for depiction of patients in the disease control group).

### B cell depletion therapy significantly induces DKK1 gene expression in skin

**Fig. 3:**
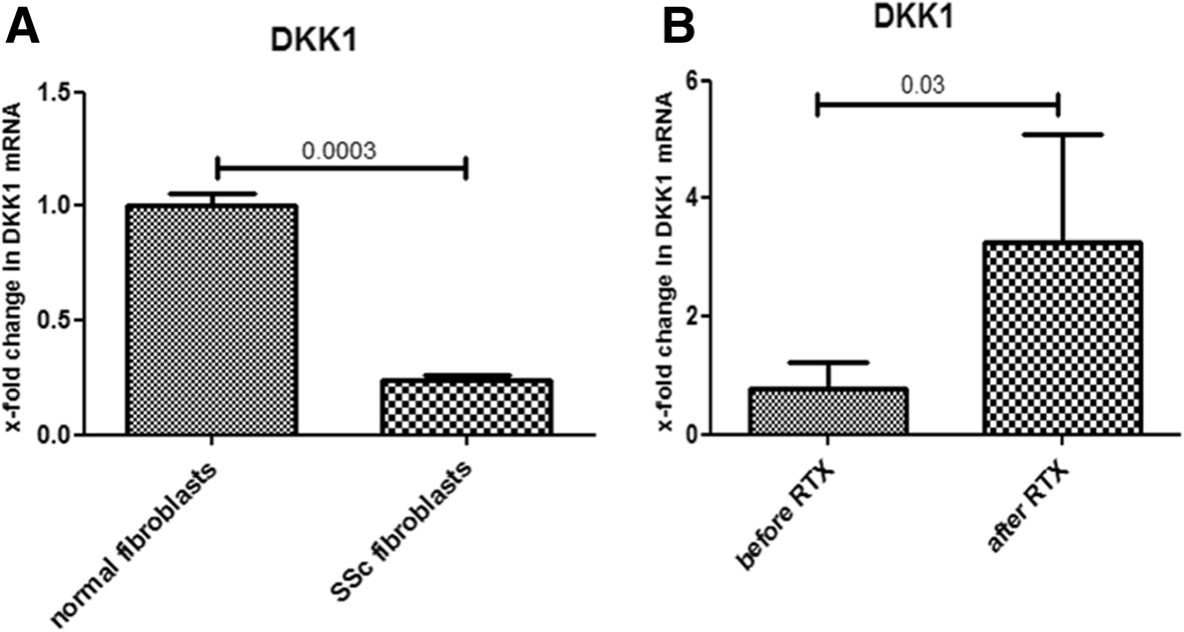
Dickkopf-1 (*DKK1*) gene is downregulated in scleoderma fibroblasts. Rituximab (*RTX*) treatment upregulates DKK1 gene expression in the skin. Fibroblasts from patients with systemic sclerosis had 4.1-fold downregulation of DKK1 gene expression compared to normal fibroblasts (*p* < 0.001) (**a**). RTX treatment mediates significant 4.2-fold upregulation of skin DKK1 gene expression (*p* = 0.030) (**b**)

We first evaluated the expression of the DKK1 gene in scleroderma compared to normal fibroblasts. There was significant 4.1-fold downregulation of DKK1 expression in fibroblasts from patients with scleroderma compared to normal fibroblasts (*p* < 0.001) as shown in Fig. 3a. We next assessed whether B cell depletion therapy affects the expression of the DKK1 gene in the skin. To address this question we analyzed DKK1 gene expression in fibroblasts extracted from skin biopsies from three patients with SSc treated with RTX (at baseline and 6 months after treatment). All three patients responded clinically with a significant decline of >30 % in the MRSS. There was significant 4.2-fold upregulation of DKK1 expression following RTX treatment (*p* = 0.030) as shown in Fig. 3b.

### Levels of Dkk-1 and TGFβ expression in skin from patients with SSc are inversely correlated

**Fig. 4:**
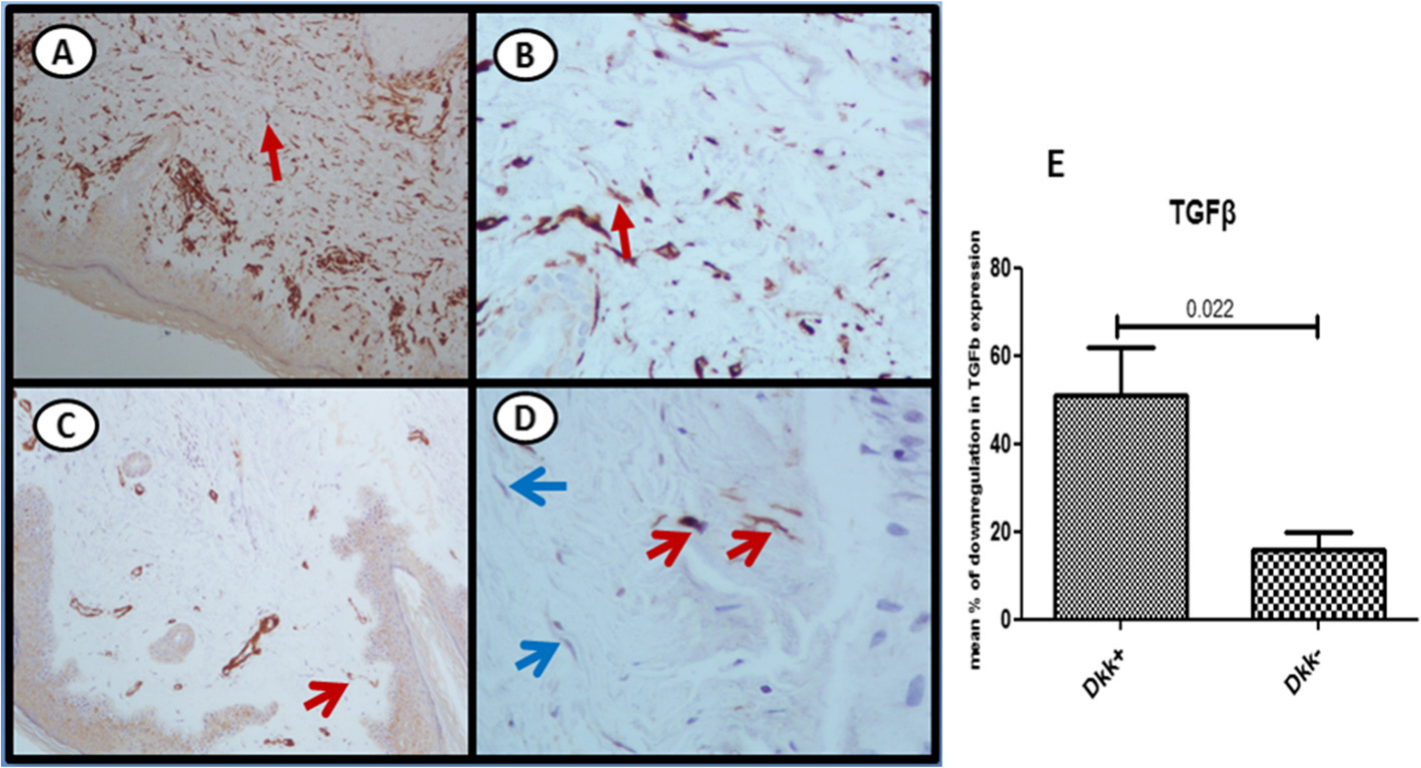
Rituximab (RTX) treatment correlates with significant attenuation of transforming growth factor β (*TGFβ*) expression in the skin. TGFβ is strongly expressed in the vast majority of spindle-like cells at baseline (*red arrows*) (**a**, **b**). Following RTX treatment significant attenuation of TGFβ expression is seen; many spindle-like cells do not express TGFβ (*blue arrows*) (**c**, **d**). Streptavidin biotin peroxidase: magnification × 20 (**a**, **c**), × 400 (**b**, **d**). Downregulation of TGFβ expression is more pronounced in the subgroup of patients with upregulation of Dkk-1 following RTX treatment (*Dkk+*) compared to the subgroup of patients who did not have upregulation of Dkk-1 following RTX treatment (*Dkk-*) (**e**)

Taking into account recently reported data indicating that TGFβ regulates Dkk-1 expression, we next asked whether TGFβ skin expression is modified following B cell depletion therapy. In skin, TGFβ expression (assessed immunohistochemically) in fibroblasts from the upper dermis was significantly attenuated following RTX treatment (mean ± SEM 32.72 ± 4.67 vs 20.21 ± 3.08, at baseline and at 6 months, respectively, *p =* 0.01). However, the downregulation of TGFβ expression in skin was significantly more pronounced in the subgroup of patients (*n* = 4) with upregulation of Dkk-1. More specifically, in this subgroup TGFβ was downregulated by a mean percentage (± SEM) of 50.88 % (±10.81) in sharp contrast to only 15.98 % (±3.73) in patients with no upregulation of Dkk-1 (*p* = 0.02) (Fig. 4e). Representative histological analysis from a patient with significant downregulation of TGFβ expression in skin is also shown in Fig. 4a-d. In the disease control group there was no change in TGFβ expression in the upper dermis (mean ± SEM 27.37 ± 6.76 vs 29.0 ± 2.15, at baseline and at 6 months, respectively, *p* value not significant). There were no changes in TGFβ expression in the lower dermis in either patient group. We further analyzed the expression of THBS1, a well-known target of TGFβ, in skin fibroblasts obtained from two patients with SSc prior to and 6 months after treatment with RTX. THBS1 expression was downregulated (by 37.93 % and 50.90 %, respectively) in both patients. Collectively these data indicate that B cell depletion therapy is associated with downregulation of TGFβ expression in skin; this downregulation is more pronounced in the subset of SSc patients with upregulation of Dkk-1.

### Upregulation of Dkk-1 in skin is associated with enhanced resolution of skin fibrosis

**Fig. 5:**
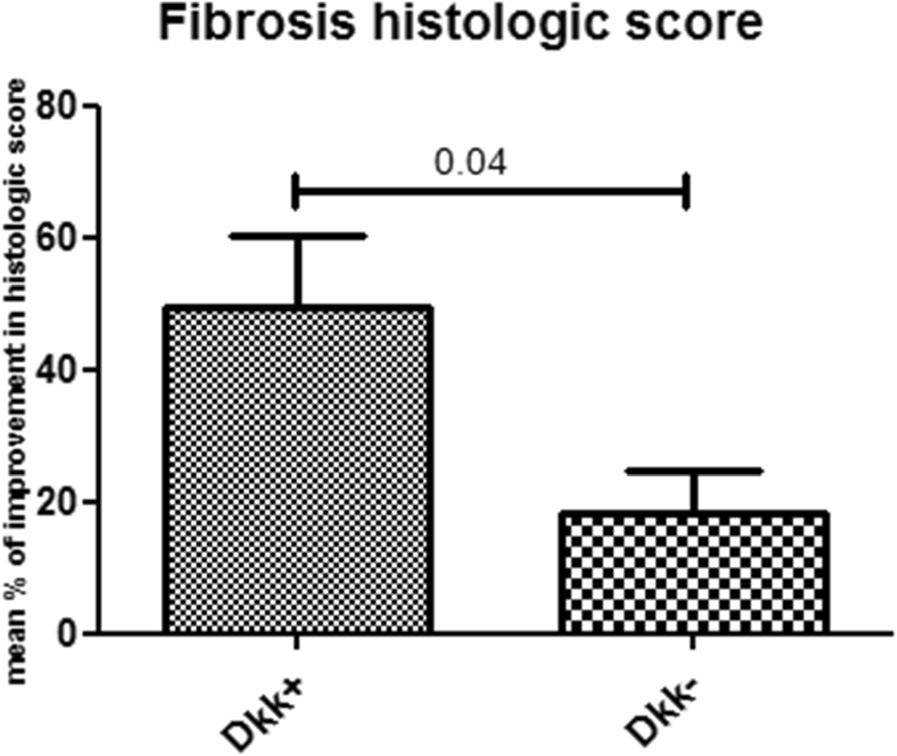
Upregulation of Dickkopf-1 (*Dkk-1*) in skin is associated with enhanced resolution of skin fibrosis. Dkk-1 upregulation following rituximab treatment is associated with a significant decrease in collagen accumulation in the upper dermis compared to patients who did not have upregulation of Dkk-1 (*p* = 0.040)

We further explored potential differences in histologic response between patients who had and those who did not have upregulation of Dkk-1. Patients with upregulation of Dkk-1 skin expression (*n* = 4) had an enhanced histologic response in the resolution of skin fibrosis. More specifically, on histologic analysis of skin from these patients, Dkk-1 upregulation was associated with a significant decrease in collagen accumulation in the upper dermis by a mean ± SEM 49.47 ± 10.63 %, compared to 18.18 ± 6.67 % in patients who did not have upregulation of Dkk-1 (*p* = 0.04) (Fig. 5). Histologic data matched the clinical data; the MRSS at 1 year (following two cycles of RTX treatment at baseline and at 6 months) decreased by a median of 63.33 % (24.34–72.92) in the subgroup with Dkk-1 upregulation compared to only 28.08 % (27.35–44.64) in the subgroup with undetectable Dkk-1; however this was not statistically significant. PFTs improved following RTX treatment, irrespective of Dkk-1 expression in skin.

### B cell depletion in skin is associated with Dkk-1 upregulation following RTX treatment

**Fig. 6:**
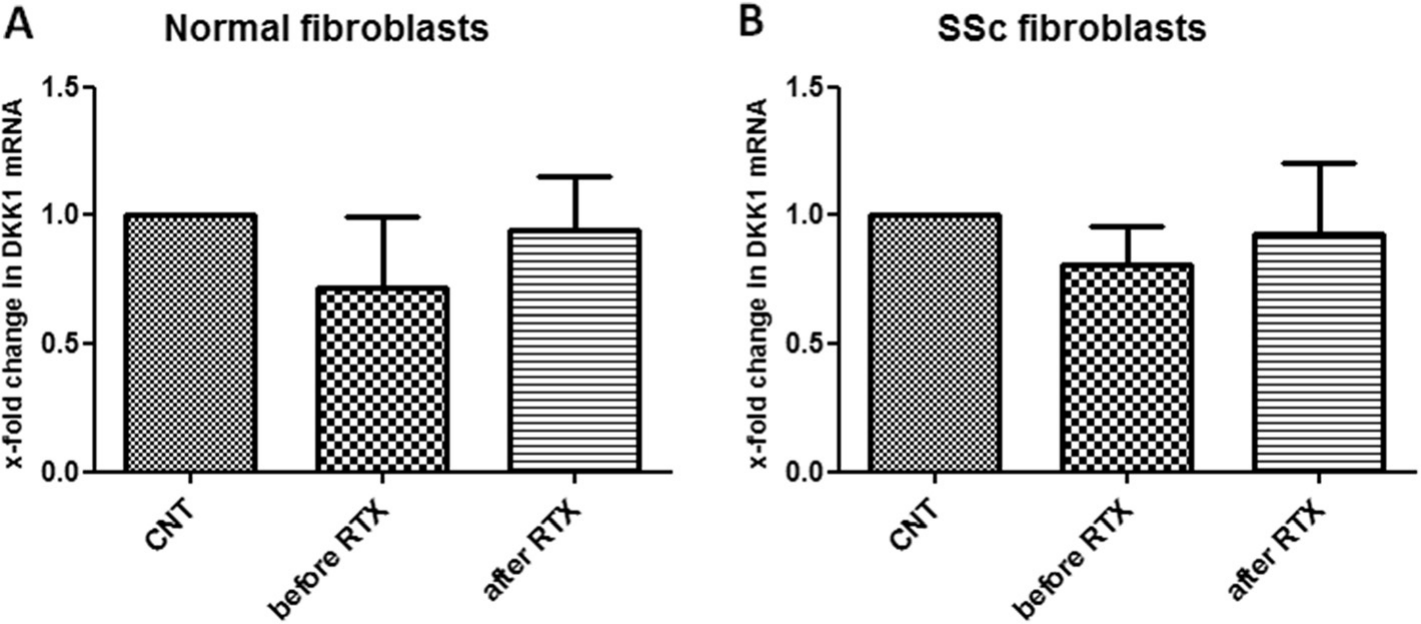
Circulating factors do not participate in Dickkopf-1 (*DKK1*) gene upregulation following rituximab (*RTX*) treatment. DKK1 gene expression in fibroblasts treated with serum obtained from patients with systemic sclerosis (*SSc*) prior to RTX treatment was similar to DKK1 gene expression in fibroblasts treated with serum obtained from patients with SSc following RTX treatment: **a** normal fibroblasts; **b** fibroblasts from patients with SSc

We next explored potential mechanisms of DKK1 upregulation in the skin following RTX treatment. We first aimed to explore whether circulating factors participate; to do so we assessed circulating levels of Dkk-1, TGFβ and IL-6 prior to and following B cell depletion therapy. Circulating levels of Dkk-1 did not change following treatment (mean ± SEM optical density (OD) 0.17 ± 0.04 vs 0.19 ± 0.03, prior to and following treatment, respectively, *p* value not significant). Serum TGFβ levels remained unchanged (mean ± SEM OD: 2.01 ± 0.38 vs 2.41 ± 0.36, prior to and following treatment respectively, *p* value not significant). This was also true for IL-6 levels (mean ± SEM OD 0.35 ± 0.07 vs 0.37 ± 0.11, prior to and following treatment, respectively, *p* value not significant). To assess whether other circulating factors may mediate DKK1 upregulation we treated normal fibroblasts and fibroblasts from patients with scleroderma with serum obtained from patients with SSc (*n* = 3) prior to and 6 months after RTX treatment. DKK1 gene expression in fibroblasts treated with serum obtained from patients with SSc prior to RTX treatment was similar to DKK1 gene expression in fibroblasts treated with serum obtained from SSc patients after RTX treatment, as shown in Fig. 6a (normal fibroblasts) and 6b (fibroblasts from patients with SSc).

We further assessed whether skin B cell depletion may correlate with DKK1 upregulation. Skin-infiltrating B cells were present in all patients with SSc in relatively small numbers; details have been previously reported [3, 5]. Briefly, RTX treatment effectively depleted skin-infiltrating B cells in four out of eight patients assessed. In the subgroup of patients who had Dkk-1 upregulation following RTX treatment (*n* = 4); three patients also had effective B cell depletion in the skin. In sharp contrast, in the subgroup of patients who did not have upregulation of Dkk-1 (*n* = 4), effective B cell depletion in the skin was evident in one patient only. These data suggest a potential link between skin B cell depletion and Dkk-1 upregulation.

## Discussion

Even though clinical data pointing to the direction of a beneficial role of RTX in SSc continue to emerge, the critical question of how B cell depletion therapy may mediate its antifibrotic effects remains largely unanswered. In animal models of SSc, B cells exhibit a disturbed phenotype by displaying increased CD19 signaling and hyper-responsiveness [23]. There is strong evidence that RTX effectively ameliorates collagen accumulation in these models [13], indicating a link between B cells and the fibrotic process.

Evidence from patients with SSc indicate that B cells are present locally in fibrotic tissue in both skin [24] and lung [25]; most importantly, gene expression analysis has revealed a B cell signature in the skin in scleroderma [26]. Even though these data suggests that B cells are active players in fibrosis, the exact mechanisms involved are not entirely known. In order to reveal these mechanisms one must explore how B cells may directly or indirectly interact with fibroblasts, the cell type responsible for collagen overproduction.

There are three potential ways whereby B cells may interact with fibroblasts [27]. First, B cells may produce agonistic fibroblast-stimulating auto-antibodies (Abs), such as anti-platelet-derived growth factor receptor (PDGFR) Abs [28]. A second way is by producing soluble mediators; B cells can produce TGFβ and IL-6, which are cytokines strongly involved in the pathophysiology of fibrosis. Finally, recent evidence suggests that B cells can stimulate fibroblasts in vitro via a contact-dependent mechanism. Francois et al. have shown that when scleroderma fibroblasts are co-cultured with B cells, there is significant upregulation of collagen production [29]. Interestingly, the effect of B cells on collagen production in this experimental model was comparable to that of TGFβ, one of the most important profibrotic molecules known. Moreover, it was shown that the effect of B cells on fibroblasts in this experimental model is contact-dependent and at least partially mediated by TGFβ.

In this study we provide experimental evidence, at both protein and gene expression level, that RTX treatment may affect Dkk-1 skin expression in patients with SSc. This is the first study to suggest a link between B cell depletion and Dkk-1 expression in skin. Dkk-1 is strikingly absent from the skin in scleroderma; however, in a subset of patients with SSc, this molecule is upregulated following RTX treatment. More importantly, the patients with Dkk-1 upregulation have the more profound histologic response to B cell depletion therapy. Our data indicate that the upregulation of Dkk-1 may represent a specific effect of RTX treatment, as it was not observed in patients with SSc who were receiving standard treatment including cyclophosphamide; however, our results should be interpreted with caution, taking into account the limited number of patients assessed. These data reinforce existing evidence that Dkk-1 is a crucial mediator of the fibrotic process.

A critical question is how RTX may affect the expression of Dkk-1 in skin. It is currently unknown whether RTX mediates its potential antifibrotic effects by depleting B cells or by other mechanisms; it is known that RTX has a broad effect on the immune system [30]. In this study we found a strong association between skin B cell depletion and Dkk-1 upregulation/histologic response. This strong association suggests that B cells in skin, or other, so far unknown factor(s), may potentially suppress Dkk-1 expression in fibroblasts and therefore, effective B cell depletion in skin alleviates this suppression, leading to Dkk-1 upregulation. If indeed this is true and B cells are responsible for the striking lack of Dkk-1 expression in fibroblasts from patients with scleroderma, the next question is how B cells mediate this effect. Our data suggest that this may be a TGFβ-dependent effect. It is also of interest that circulating levels of Dkk-1 and TGFβ did not change following treatment, indicating that the whole process is taking place in the affected tissues such as the skin; this is in accordance with previously reported data [31].

Our study has several potential limitations. The first one is the relatively small number of patients assessed, therefore, definite conclusions cannot be drawn. The second limitation is that the expression of TGFβ in skin was assessed mainly by immunohistochemical analysis, which cannot distinguish between active and latent forms of TGFβ. Therefore, the evidence provided in our study implicating TGFβ in the regulation of Dkk-1 expression should not be considered powerful. Further studies are needed to clarify the potential role of TGFβ in this process. Moreover, an additional limitation is that Wnt pathway activation in the skin was not assessed.

## Conclusions

This is the first study that demonstrates a link between B cell depletion and upregulation of Dkk-1 in the skin of patients with SSc. Moreover, patients with upregulation of Dkk-1 following RTX treatment have the best histologic response to treatment. Large-scale randomized controlled studies assessing the efficacy of RTX in SSc are highly needed.

## Additional files

Additional file 1: Figures S1-S3. In the baseline biopsy (**A** and **B**) Dkk-1 is not expressed in epidermis (*black arrow*), appendices (*green arrow*) and spindle-like cells (*red arrows*). In the follow up biopsy (**C** and **D**) there is clear expression of Dkk-1 in epidermis (*black arrow*), appendices (*green arrow*) and spindle-like cells (*red arrows*). All three patients responded to RTX treatment. Streptavidin biotin peroxidase (**A** and **D** × 20, **B** and **E** × 400) (ZIP 5323 kb) Additional file 2: Figures S4-S6. Dkk-1 is not expressed in epidermis (*black arrow*), appendices (*green arrow*) and spindle-like cells (*red arrows*) prior to (**A** and **B**) and following RTX treatment (**D** and **E**). These patients did not respond to RTX treatment. Streptavidin biotin peroxidase (**A** and **D** × 20, **B** and **E** × 400) (ZIP 5591 kb) Additional file 3: Figure S7. Dkk-1 is not expressed in epidermis (*black arrow*), appendices (*green arrow*) and spindle-like cells (*red arrows*) in both baseline (**A** and **B**) and follow up biopsy (**D** and **E**) in a non responder. Streptavidin biotin peroxidase (**A** and **D** × 20, **B** and **E** x400). RTX treatment had no effect on collagen accumulation (**C** and **F**, prior to and following RTX treatment, respectively). Masson’s trichrome × 100 (TIF 876 kb) Additional file 4: Figures S8-S10. Dkk-1 is not expressed in epidermis (*black arrow*), appendices (*green arrow*) and spindle-like cells (*red arrows*) in the control patient group at baseline (**A** and **B**) and follow up biopsies (**D** and **E**). Streptavidin biotin peroxidase (**A** and **D** × 20, **B** and **E** × 400) (ZIP 5018 kb)

## Abbreviations

Dkk-1: Dickkopf-1
DLCO: diffusing lung capacity for carbon monoxide
FBS: fetal bovine serum
FVC: forced vital capacity
GAPDH: glyceraldehyde 3-phosphate dehydrogenase
IL-6: intrleukin-6
MRSS: modified Rodnan skin score
OD: optical density
PDGFR: platelet-derived growth factor receptor
PFT: pulmonary function test
RTX: rituximab
SEM: standard error of the mean
SSc: systemic sclerosis
TFGβ: transforming growth factor β
