# Supplementary figures and images for: B cell depletion therapy upregulates Dkk-1 skin expression in patients with systemic sclerosis: association with enhanced resolution of skin fibrosis
Source: Arthritis Res Ther. 2016 May 21;18:118. doi: 10.1186/s13075-016-1017-y (PMC4875588; doi:10.1186/s13075-016-1017-y)

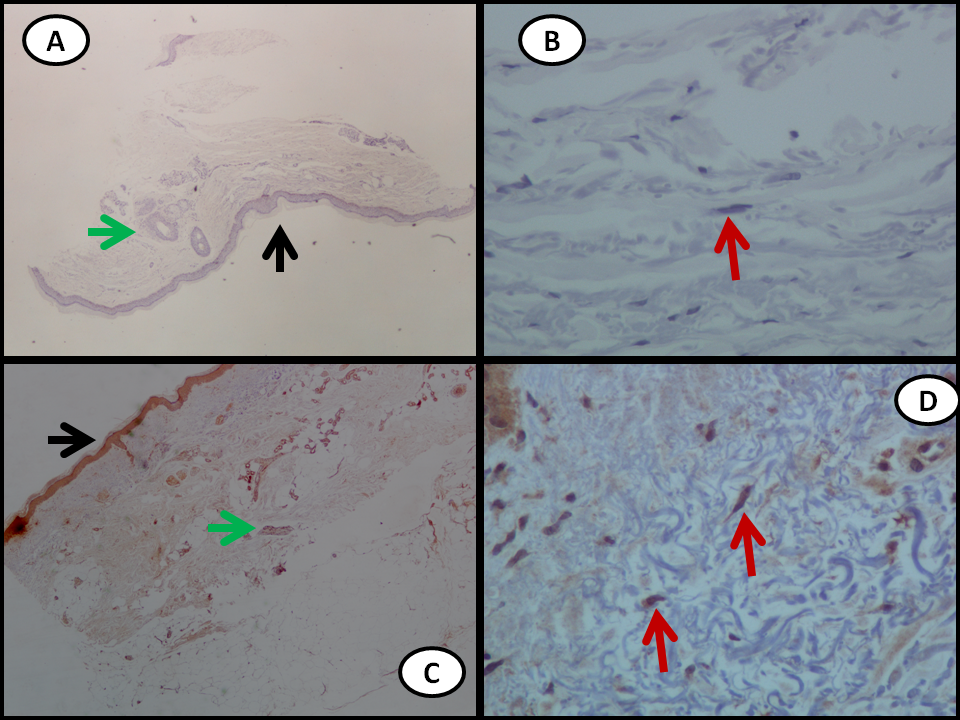

Supplement: Additional file 1: Figures S1-S3. — In the baseline biopsy (A and B) Dkk-1 is not expressed in epidermis (black arrow), appendices (green arrow) and spindle-like cells (red arrows). In the follow up biopsy (C and D) there is clear expression of Dkk-1 in epidermis (black arrow), appendices (green arrow) and spindle-like cells (red arrows). All three patients responded to RTX treatment. Streptavidin biotin peroxidase (A and D × 20, B and E × 400) (ZIP 5323 kb) [file 13075_2016_1017_MOESM1_ESM.zip › Sup Figure 1_RTX-Dkk+.tif]

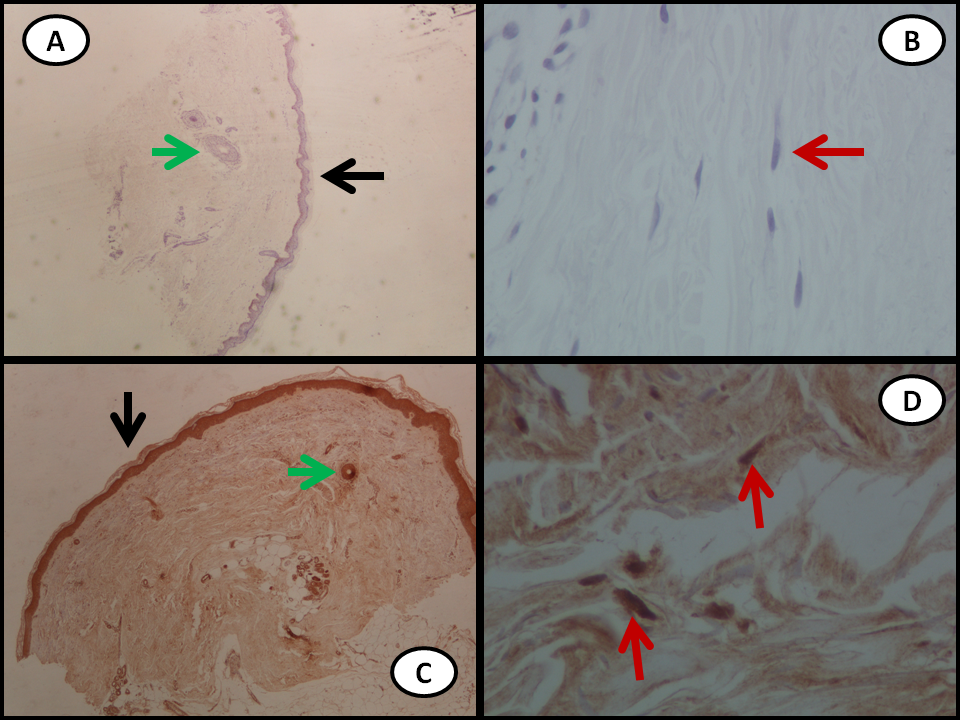

Supplement: Additional file 1: Figures S1-S3. — In the baseline biopsy (A and B) Dkk-1 is not expressed in epidermis (black arrow), appendices (green arrow) and spindle-like cells (red arrows). In the follow up biopsy (C and D) there is clear expression of Dkk-1 in epidermis (black arrow), appendices (green arrow) and spindle-like cells (red arrows). All three patients responded to RTX treatment. Streptavidin biotin peroxidase (A and D × 20, B and E × 400) (ZIP 5323 kb) [file 13075_2016_1017_MOESM1_ESM.zip › Sup Figure 2_RTX-Dkk+.tif]

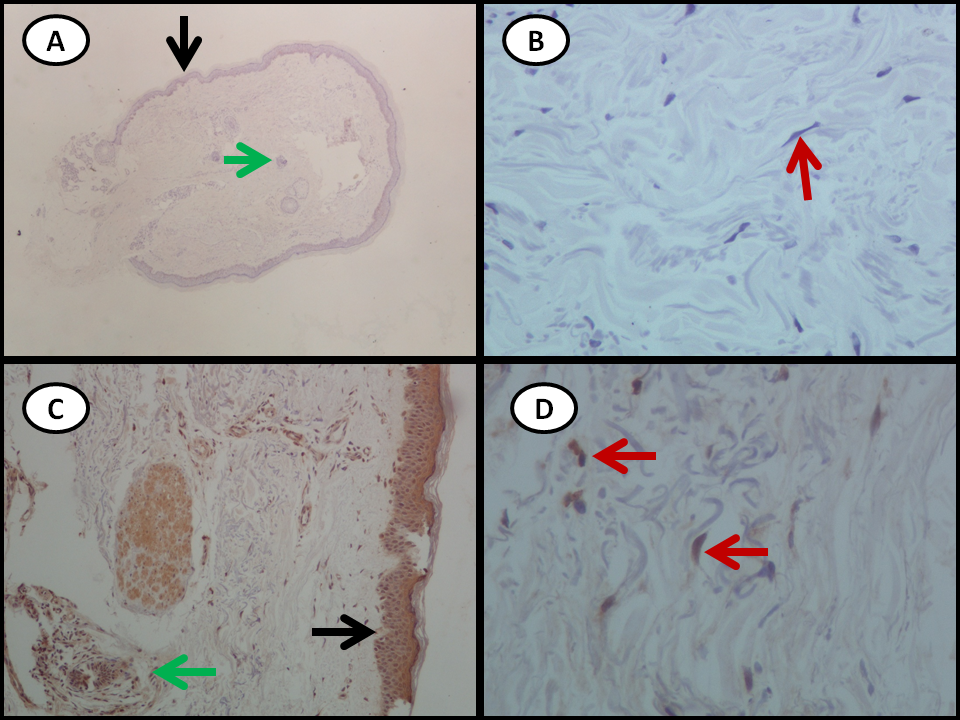

Supplement: Additional file 1: Figures S1-S3. — In the baseline biopsy (A and B) Dkk-1 is not expressed in epidermis (black arrow), appendices (green arrow) and spindle-like cells (red arrows). In the follow up biopsy (C and D) there is clear expression of Dkk-1 in epidermis (black arrow), appendices (green arrow) and spindle-like cells (red arrows). All three patients responded to RTX treatment. Streptavidin biotin peroxidase (A and D × 20, B and E × 400) (ZIP 5323 kb) [file 13075_2016_1017_MOESM1_ESM.zip › Sup Figure 3_RTX-Dkk+.tif]

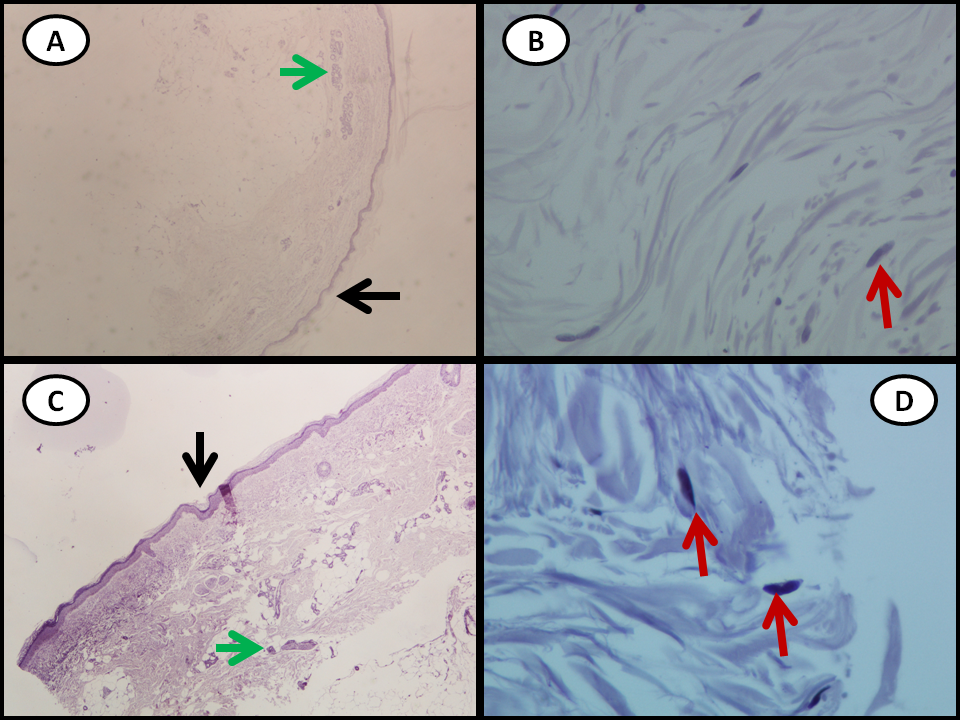

Supplement: Additional file 2: Figures S4-S6. — Dkk-1 is not expressed in epidermis (black arrow), appendices (green arrow) and spindle-like cells (red arrows) prior to (A and B) and following RTX treatment (D and E). These patients did not respond to RTX treatment. Streptavidin biotin peroxidase (A and D × 20, B and E × 400) (ZIP 5591 kb) [file 13075_2016_1017_MOESM2_ESM.zip › Sup Figure 4_RTX_Dkk-.tif]

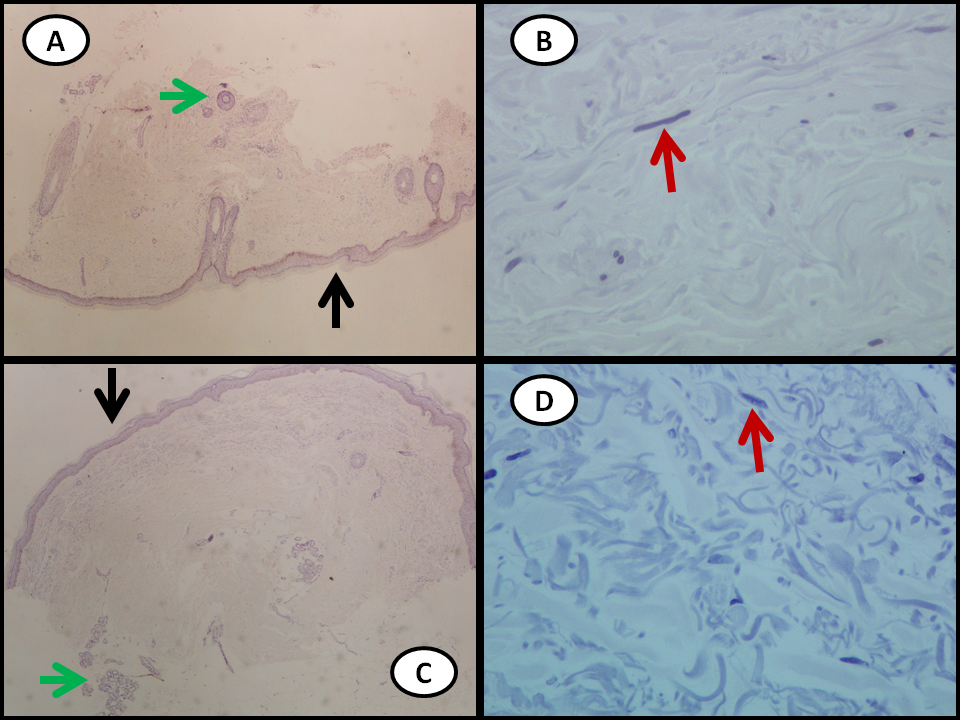

Supplement: Additional file 2: Figures S4-S6. — Dkk-1 is not expressed in epidermis (black arrow), appendices (green arrow) and spindle-like cells (red arrows) prior to (A and B) and following RTX treatment (D and E). These patients did not respond to RTX treatment. Streptavidin biotin peroxidase (A and D × 20, B and E × 400) (ZIP 5591 kb) [file 13075_2016_1017_MOESM2_ESM.zip › Sup Figure 5_RTX_Dkk-.tif]

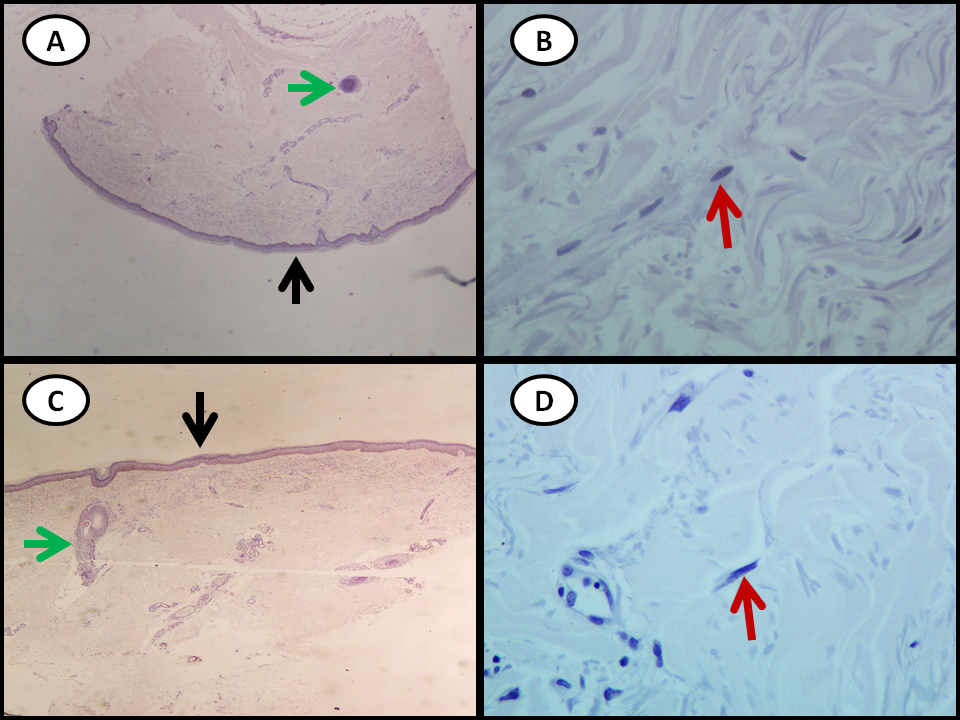

Supplement: Additional file 2: Figures S4-S6. — Dkk-1 is not expressed in epidermis (black arrow), appendices (green arrow) and spindle-like cells (red arrows) prior to (A and B) and following RTX treatment (D and E). These patients did not respond to RTX treatment. Streptavidin biotin peroxidase (A and D × 20, B and E × 400) (ZIP 5591 kb) [file 13075_2016_1017_MOESM2_ESM.zip › Sup Figure 6_RTX-Dkk-.tif]

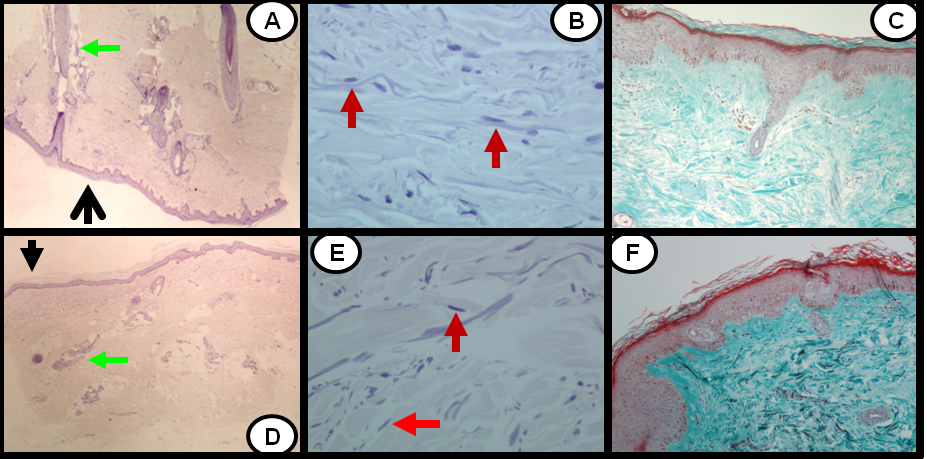

Supplement: Additional file 3: Figure S7. — Dkk-1 is not expressed in epidermis (black arrow), appendices (green arrow) and spindle-like cells (red arrows) in both baseline (A and B) and follow up biopsy (D and E) in a non responder. Streptavidin biotin peroxidase (A and D × 20, B and E x400). RTX treatment had no effect on collagen accumulation (C and F, prior to and following RTX treatment, respectively). Masson’s trichrome × 100 (TIF 876 kb) [file 13075_2016_1017_MOESM3_ESM.tif]

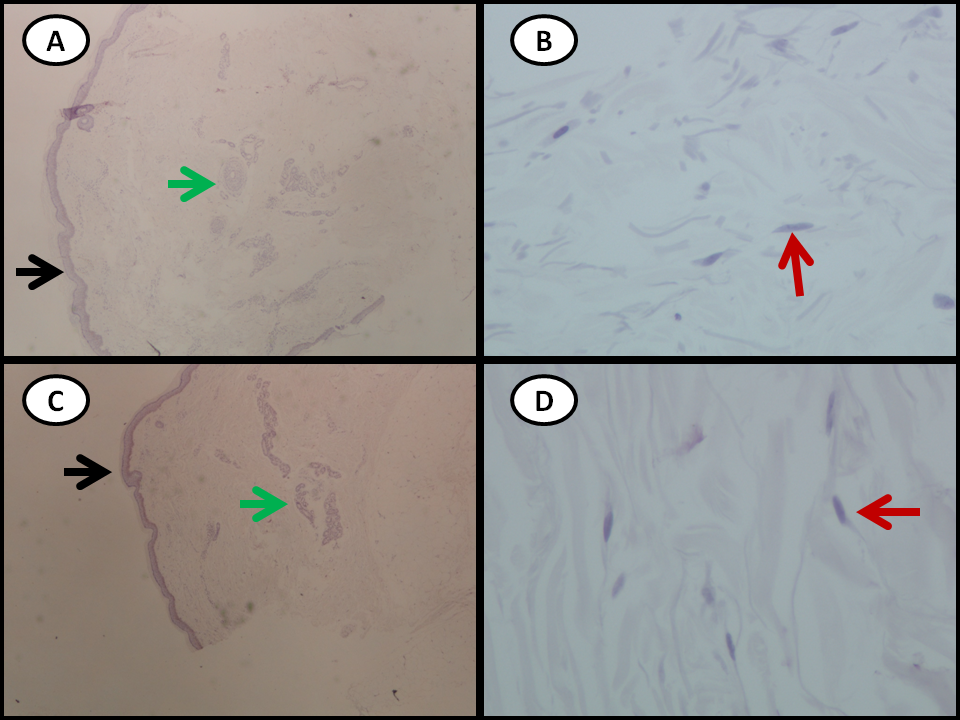

Supplement: Additional file 4: Figures S8-S10. — Dkk-1 is not expressed in epidermis (black arrow), appendices (green arrow) and spindle-like cells (red arrows) in the control patient group at baseline (A and B) and follow up biopsies (D and E). Streptavidin biotin peroxidase (A and D × 20, B and E × 400) (ZIP 5018 kb) [file 13075_2016_1017_MOESM4_ESM.zip › Sup Figure 10_SSc_Cnt3.tif]

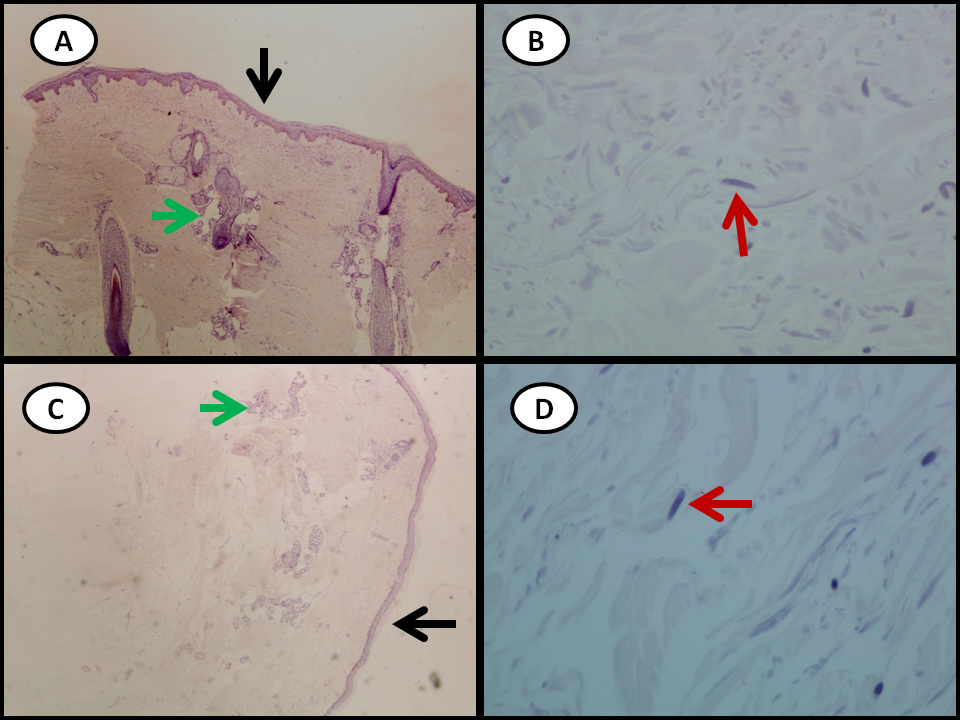

Supplement: Additional file 4: Figures S8-S10. — Dkk-1 is not expressed in epidermis (black arrow), appendices (green arrow) and spindle-like cells (red arrows) in the control patient group at baseline (A and B) and follow up biopsies (D and E). Streptavidin biotin peroxidase (A and D × 20, B and E × 400) (ZIP 5018 kb) [file 13075_2016_1017_MOESM4_ESM.zip › Sup Figure 8_SSc_Cnt1.tif]

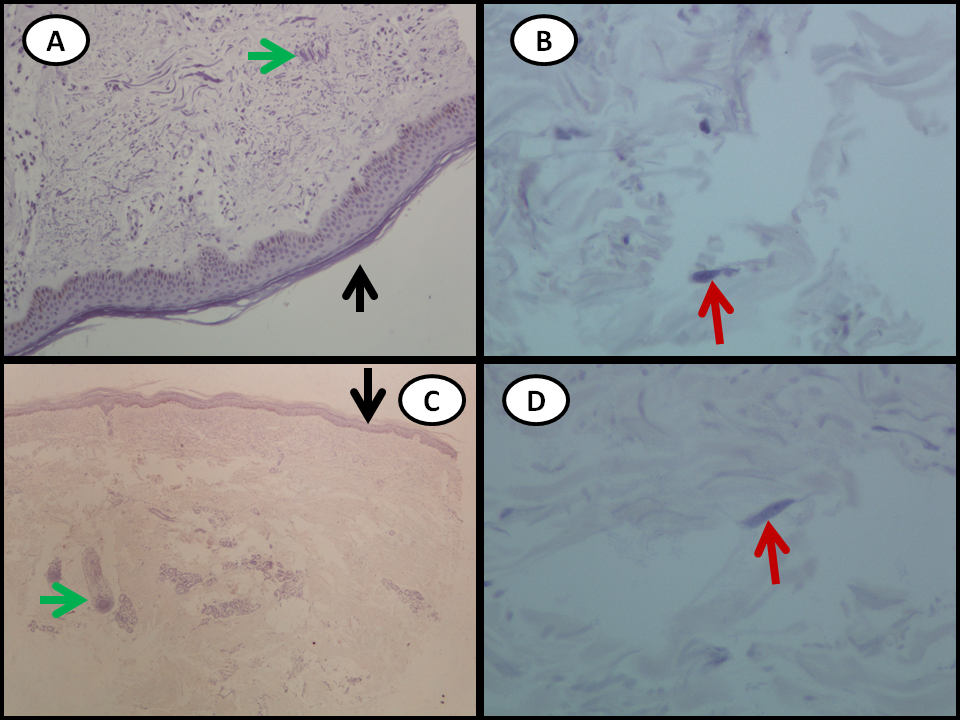

Supplement: Additional file 4: Figures S8-S10. — Dkk-1 is not expressed in epidermis (black arrow), appendices (green arrow) and spindle-like cells (red arrows) in the control patient group at baseline (A and B) and follow up biopsies (D and E). Streptavidin biotin peroxidase (A and D × 20, B and E × 400) (ZIP 5018 kb) [file 13075_2016_1017_MOESM4_ESM.zip › Sup Figure 9-SSc_Cnt2.tif]
